# Assessing social structure: a data-driven approach to define associations between individuals

**DOI:** 10.1007/s42991-022-00231-9

**Published:** 2022-03-25

**Authors:** Sara B. Tavares, Hal Whitehead, Thomas Doniol-Valcroze

**Affiliations:** 1 Cetacean Research Program, Pacific Biological Station, Fisheries and Oceans Canada, Nanaimo, Canada; 2 Department of Biology, Dalhousie University, Halifax, Canada

**Keywords:** Association, Lagged identification rate, Social structure, Northern resident killer whales, *Orcinus orca*

## Abstract

Our interpretation of animal social structures is inherently dependent on our ability to define association criteria that are biologically meaningful. However, association thresholds are often based upon generalized preconceptions of a species’ social behaviour, and the impact of using these arbitrary definitions has been largely overlooked. In this study we suggest a probability-based method for defining association thresholds using lagged identification rates on photographic records of identifiable individuals. This technique uses a simple model of emigration/immigration from photographable clusters to identify the time-dependent lag value between identifications of two individuals that corresponds to approximately 75% probability of being in close spatial proximity and likely associating. This lag value is then used as the threshold to define associations for social analyses. We applied the technique to a dataset of northern resident killer whales (*Orcinus orca*) in the Northeast Pacific and tested its performance against two arbitrary thresholds. The probabilistic association maximized the variation in association strengths at different levels of the social structure, in line with known social patterns in this population. Furthermore, variability in inferred social structure metrics generated by different association criteria highlighted the consequential effect of choosing arbitrary thresholds. Data-driven association thresholds are a promising approach to study populations without the need to subjectively define associations in the field, especially in societies with prominent fission–fusion dynamics. This method is applicable to any dataset of sequential identifications where it can be assumed that associated individuals will tend to be identified in close proximity.

**Supplementary Information:**

The online version contains supplementary material available at 10.1007/s42991-022-00231-9.

## Introduction

The social structure of a population delineates ecological relationships between conspecifics and the circumstances under which communication takes place (Whitehead 1997). An understanding of animal social structure is vital for management and conservation work (Snijders et al. 2017), understanding the evolution of social and other traits (e.g., Lehmann et al. 2007; Ellis et al. 2017) as well as information flow and disease transmission (e.g., Cross et al. 2004; Hasenjager and Dugatkin 2016). Using Hinde’s (1976) conceptual framework, social structure is studied in a bottom-up approach, by quantifying how individuals interact with one another, to understand their relationships and subsequently the whole social structure, often by recording interactions or associations among individually identified animals (e.g., Bigg et al. 1990; Whitehead et al. 1991; Wittemyer et al. 2005). Interactions may be difficult or impossible to observe (e.g., for nocturnal animals or individuals that live underground or underwater) and associations (i.e., circumstances in which interactions can take place) are commonly used as a proxy for interactions instead (Whitehead 1997, 2008a; Farine 2015). Therefore, our capacity to correctly discern associations and define association thresholds will impact the perceived social structure of a population, since a threshold that is too broad may include dyads that are not truly associating but a narrow one may omit true associations (Whitehead 2008a).

Associations are often recorded by identifying dyads of individuals within a temporal and/or spatial threshold that denotes proximity (e.g., sperm whales, *Physeter microcephalus*, photographed within 2 h of each other, Whitehead et al. 1991; spotted wobbegong sharks, *Orectolobus maculatus*, within 4 m of each other, Armansin et al. 2016), sometimes in combination with behavioural state uniformity between two individuals (e.g., humpback whales, *Megaptera novaeangliae*, up to 2 body lengths apart and displaying coordinated behaviour, Weinrich 1991), or several dyads within pre-defined group boundaries (e.g., all southern resident killer whales, *Orcinus orca*, within 3 body lengths of each other, Ellis et al. 2017; Rothschild’s giraffes, *Giraffa camelopardalis rothschildi*, within 1 km, Muller et al. 2018). Defining the threshold for associations can be quite challenging (Whitehead 1995; Whitehead and Dufault 1999; Gowans et al. 2007) and there is no standard procedure for this, being often arbitrarily decided or following practices from other studies. Furthermore, while some association criteria can be unambiguously measured by observers, others might not be easily measured in the field, especially across different behavioural contexts or across populations/species with different social dynamics and with grouping patterns that are not equally discernible (e.g., fish-eating killer whales in Iceland versus [vs.] in the Northeast Pacific: Bigg et al. 1990; Tavares et al. 2017; dusky dolphins, *Lagenorhynchus obscurus*, in Argentina vs. in New Zealand: Markowitz 2004; Würsig and Würsig 1980).

To reduce arbitrariness in the choice of association parameters, recent studies have been focussing on data-driven approaches to define associations among conspecifics. For example, Psorakis et al. (2015) used the properties of the data stream of timestamped information on individual usage of feeding locations by great tits, *Parus major*, from passive integrated transponders (PIT) tag-based data, to find periods of increased bird visitations to a feeder. The study used Gaussian mixture models to cluster these into non-overlapping gathering events within each of which individuals present were all considered associated. Tavares et al. (2017) conducted dedicated fieldwork to collect timestamped digital photographs of Icelandic killer whales and used a maximum-likelihood estimation of the length of photographic bouts to derive the time period within which two photographed individuals were considered associated for the day. Data-driven methods, such as these, often need large samples of precise timestamped data collected using standardised protocols. However, such data are not always available (especially when social analyses are applied to existing or archival datasets). Thus, there is a need for methods to quantify associations that can be applied without having to assess behavioural criteria in-situ, and for data that have already been collected for a purpose other than social analysis.

**Fig. 1:**
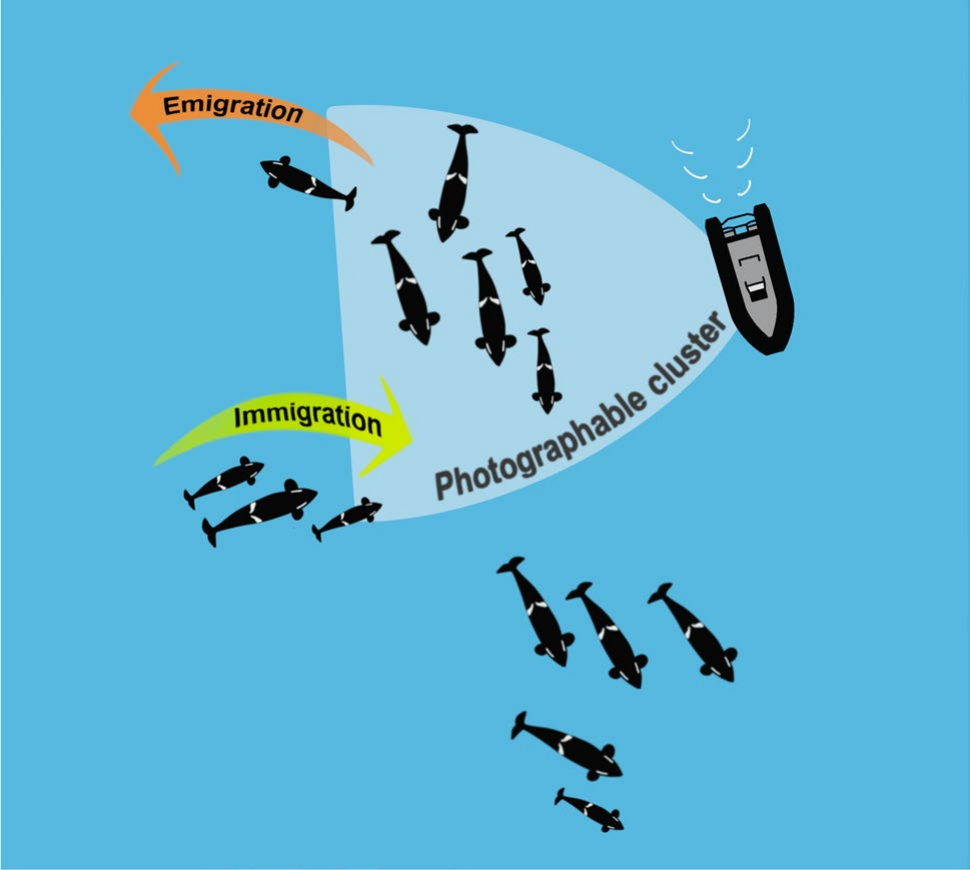
Schematic of the data collection process illustrating the terms relevant for this method. Individuals surfacing together are approached by the research boat; individuals together in a photographable cluster (light blue shaded area), visible to the photographer, are photographed in close succession to one another. Throughout the data collection event, individuals can move out of the photographable cluster (emigration), even if they remain in the study area, or move into the photographable cluster (immigration). Note that emigration to and immigration from the photographable cluster can either happen actively, due to the animals’ behaviour, or passively, due to changes in the photographers’ location (e.g., boat leaving one grouping to approach another in a different section of the study area)

Here, we present a data-driven method for defining association in different forms of social data and, for clarity, we illustrate the concepts of this method on photo-identification data. The basis of this methodology is that if two individuals are identified some unit of time apart, the probability that they were associated declines in relation to the amount of time individuals remain in the area in which the identifications are collected. This decline can be quantified by the lagged identification rate (Whitehead 2001). Typically, the lagged identification rate is used to investigate how animals move into, and out of, a study area. However, we propose adapting this metric at a shorter time scale, to investigate how animals move into, and out of, identification range during a sampling period (Fig. 1). By modelling the changes in group presence within the range of the observer, we can derive the cut-off value for when associations are likely to take place. The main assumptions of this methodology are that individuals identified close together in time are also close in space, and that animals that prefer to associate will be identified together or in close proximity more often.

As an empirical example, we applied this method to photographic identifications of northern resident killer whales off the coast of British Columbia (Canada), where individuals can be identified by the shape and natural markings of the dorsal fin and saddle patch (Bigg 1982). Northern resident killer whales are known to have a stable society based upon long-term kinship associations; females and their philopatric descendants (i.e., her offspring, of both sexes, and the descendants, of both sexes, of her female offspring) form matrilines (basic social units) that associate together the majority of time (Bigg et al. 1990; Ford et al. 2000). While individuals within matrilines associate strongly with each other (and their proximity when travelling together is largely correlated with genealogy), individuals from different matrilines will also associate, albeit less often and at varying degrees across dyads (Bigg et al. 1990; Ford and Ellis 2002). We evaluated the performance of a probabilistic association threshold at highlighting these biological features of the social system by quantifying the variety of association strengths. We compared the data-driven threshold to two arbitrary ones at the two ends of a proximity spectrum: either considering individuals were associated when identified in the same frame together or associated if identified in the same encounter (sampling event of all individuals within visual range of the observers).

Since photo-identification data have been collected for this population since the early 1970s, predominantly for census purposes (e.g., DFO 2019), the dataset comprises both film and digital photography (types of data collection systems that impose different constraints on the way data is collected), as well as data collected during both dedicated and non-dedicated efforts. This diversity of techniques allowed us to assess the performance of the method with different data types. Additionally, we compared social structure results obtained when using the probabilistic and arbitrary association thresholds on this dataset and assessed the impact of using different thresholds on our perception of the social structure.

## Methods

### Probabilistic association threshold

#### Defining association probability

Considering photographic social data, this method assumes that the dataset consists of a series of photographic identifications of individuals, along with the date, location of the identifications and at least one ordinal parameter that follows the order of the photographic collection and allows for calculation of a lag measure between identifications (e.g., time difference in seconds or photographic frame number difference). During data collection, we assumed that individuals are identified within “photographable clusters”, i.e., collections of associated individuals available to be photographed at any time (Fig. 1). The photographable cluster is a theoretical boundary, contingent to the range within which the photographer is able to collect usable photo-identifications, and individual clusters need not be distinguishable when collecting the data. Individuals can move into (“immigration”) or out of (“emigration”) a photographable cluster (Fig. 1), when the individual enters/leaves the area/cluster being photographed or the photographer moves to another cluster. These movements of individuals into/out of a photographable cluster may be imperceptible or unclear during data collection, e.g., when many individuals are present in a relatively small area.

Since we are interested in knowing which individuals were associating in close proximity when they were photographed, we calculate the probability that individuals overlapped in a photographable cluster during sampling, i.e., that both individuals were present in the same cluster of individuals at the same time. Assuming that immigration and emigration rates are approximately equal, and that relatively few photo-identifications are taken per time unit, if photographs of two different individuals are *T* time units apart and the rate of emigration from a photographable cluster is *μ* per time unit per individual, the probability that they did not overlap in a photographable cluster is given by:

1 $$P\left( {\text{no overlap}} \right) = \mathop \int \limits_{t = 0}^{T} {\text{e}}^{ - \mu t} \cdot \left( {1 - {\text{e}}^{{ - \mu \left( {T - t} \right)}} } \right) \cdot \mu \cdot {\text{d}}t,$$

where *t* is when the first individual leaves the photographable cluster. From this expression, we derive:

2 $$P\left( {\text{no overlap}} \right) = 1 - \left( {1 + \mu \cdot T} \right){\text{e}}^{ - \mu T} .$$

We assume that reimmigration into the same cluster happens over longer time scales than the ones discussed here and can therefore be disregarded. Thus, the probability that two individuals, photographed *T* time units apart, were in the same photographable cluster together at some intervening period of time (and therefore assumed to have had associated) is:

3 $$P\left( {{\text{overlap}}} \right) = (1 + \mu \cdot T) \cdot {\text{e}}^{ - \mu T} .$$

If we set *T* to the mean time individuals are present in the photographable cluster (i.e., *T* = 1/*μ*), the probability of individuals overlapping in a photographable cluster becomes 0.736. In other words, using the mean “residence time” of individuals in a photographable cluster as the threshold for defining associations ensures an almost 75% probability that individuals photographed within that period of time were together in that cluster, and thus are assumed to have been associating during a specific sampling period (e.g., day). Therefore, we define the mean time individuals are present in a photographable cluster as the “probabilistic association threshold”.

#### Calculating lagged identification rates (LIR) and model fitting

The advantage of our approach is that the mean residence time in a photographable cluster can be calculated with the lagged identification rate (LIR) proposed by Whitehead (2001). LIR estimates the probability that an individual identified in a photograph at time $t$ can be identified again at time $t + \tau$. We obtained the LIR as a fitted function using observed data by estimating, for each lag $\tau$, the proportion of pairs of identifications of the same individual that are $\tau$ units apart (Whitehead 2001).

Adapting the LIR to estimate the probabilistic association threshold requires careful selection of the ordinal parameter used to calculate lag values between identifications and the collection events within which identifications are binned. The ordinal parameter to calculate lags between identifications will often be real time, but other ordinal measures that follow the order of collection of identifications, such as photographic frame numbers, can be used as well. To define the binning of identifications, two factors need to be set. First, the parameter or combination of parameters, that define the collection events of each sequence of identifications. This could be, for example, a date parameter defining sampling days, or date and photographer, when multiple photographers collected data within a sampling day. Then, we multiply this factor by an offset value, greater than the maximum lag value within any collection event (i.e., ${\text{ordinal parameter }} + {\text{collection event }} \times {\text{ offset value}}$). This ensures that lags are only calculated among identifications within the same sequence of data collection.

Before proceeding to model fitting, the maximum lag to be considered, in the units of the ordinal parameter, should be set to the maximum lag value within any collection event (e.g., maximum number of frames taken in a sampling day). Since there will typically be several collection events, this will ensure that we are only considering lags within collection events, with no incidental values at higher lags confounding the model fitting. A mathematical model of emigration and reimmigration can then be fitted to the data, to estimate the threshold within which individuals are highly likely to be associating:

4 $$R\left( T \right) = \left( \frac{1}{a} \right)\frac{{\left( \frac{1}{c} \right) + \left( \frac{1}{b} \right)e\left( { - \left( {\frac{1}{c} + \frac{1}{b}} \right)T} \right)}}{{\frac{1}{c} + \frac{1}{b}}} ,$$

where *T* is the lag value, *a* is the number of individuals in the focus area, *b* is the mean time in the focus area and *c* is the mean time outside of it. In analyses of movements, this exponential model is typically used to calculate emigration and reimmigration rates, as well as mean residence time in a given study area (Whitehead 2001). Instead, we propose using this model to calculate the probabilistic association threshold, i.e., the mean time individuals stay in a photographable cluster (corresponding to the *b* parameter in Eq. 4).

### Evaluation of the method

#### Test dataset

The test dataset consists of photographic identifications of 344 northern resident killer whales, collected during a total of 157 days over 4 years: the last 2 years where film cameras were used exclusively for data collection (1997 and 1998) and the first 2 years where only digital cameras were used (2009 and 2010). In these 4 years, photographic data collection was either dedicated or non-dedicated. Dedicated data collection includes data collected during research effort, when conducting scientific surveys onboard of research vessels, primarily during the summer from June through mid-September, and throughout the year by someone trained for this purpose. Non-dedicated data collection includes data collected by non-trained photographers (e.g., by the public or local whale watching communities) and by photographers in research platforms not primarily doing photographic data collection (e.g., secondary photographer taking some photographs throughout a sampling period with inconsistent effort) or in passive platforms (e.g., when a survey ship was not being driven for photographic data collection).

Photographic identifications of individuals were collected during encounters. An encounter was defined as all killer whales that were within visual range of the observers. Therefore, encounter is not equivalent to a group and could be comprised by an aggregation of groups of killer whales. Encounters lasted until all whales were photographed or until data collection was halted due to weather conditions and/or lack of sufficient light. Since for each encounter there may be more than one photographer collecting data, for the purpose of this study, data collected by each photographer is considered as a different encounter (hereafter named simply as encounter; see Figs. S1–S3 in Supplementary Material for number of encounters by photographic data type). When using film photography, only the times of the start and end of the encounter were noted, and sequential frames were assigned sequential numbers (where the first frame of a subsequent roll would be assigned the following number to the last frame of the previous roll). For digital photography, sequential photographs were assigned sequential numbers and each frame includes metadata which recorded a timestamp accurate to the second. During the encounters, neither field groupings nor associations were consistently recorded since there were no established criteria (e.g., record groupings of killer whales observed behaving in a generally coordinated fashion) across encounters/observers.

#### Calculating the probabilistic association threshold

Except when noted otherwise, all analyses described below were conducted using SOCPROG 2.8 (Whitehead 2009) in MatLab 8.5 (MathWorks, Natick, MA, USA) and figures were created in R 3.6.1 (R Core Team 2019). We calculated the probabilistic association threshold applying the methodology described above to the whole dataset (i.e., all individuals included) using the photographic frame numbers as the time variable. Frame number was chosen over photographic timestamp since it was the only ordinal parameter representative of the order of the photographic collection that was constant to both film and digital photography (see Appendix for probabilistic association threshold calculation using digital photography timestamps). The LIR estimation was implemented in SOCPROG by setting the sampling period as ${\text{Frame number}} + {\text{Encounter}} \times 10,000$ to allow for frames from the same encounter to be considered separately from other encounters. An ${\text{emigration}} + {\text{reimmigration}}$ model, available in SOCPROG, where parameters represent residence times, was fitted to the data using maximum likelihood and binomial loss (Whitehead 2001, 2017). The probabilistic association threshold was subsequently extracted from the model fitting as the mean time in a photographable cluster, corresponding to parameter *b* in the fitted model (Eq. 4).

#### Calculating association indices

To reduce bias from including infrequently sighted individuals and individuals that may have died during the period of the study or were born after the first year, the calculation of association indices was restricted to the 112 individuals that were seen on at least 5 different days (range of 5–23 days, mean of 11 ± 4 days) and in both the first year (1997) and the last year (2010) used in this study. These individuals belonged to a total of 28 matrilines (i.e., group of descendants of a shared maternal ancestor that spend the great majority of time together, Bigg et al. 1990). Since matrilines are named after the most recent matriarch, the matriline memberships assigned by Bigg et al. (1990) were used in this study; in more recent years, some matrilines were renamed to depict the fact that the old matriarch had died and each daughter with descendants became the most recent matriarch of her own matriline.

Associations among the 112 individuals were calculated using the half-weight index (HWI), with day as the sampling period, for three different criteria:

1. Probabilistic association. After calculating the association threshold estimated from the data-driven method described above, we considered individuals associated for the day (sampling period) if they were photographed in the same encounter within that specific number of frames at least once during that day;
2. Same-frame criterion. Individuals were considered associated for the day (sampling period) if both were identified in the same frame at least once during that day;
3. Encounter criterion. Individuals were considered associated for the day (sampling period) if photographed in the same encounter.

#### Performance testing

We evaluated the performance of the probabilistic association threshold by investigating the efficiency of this approach to generate patterns that reflect known social aspects of the population. Northern resident killer whales have a stable society but do not associate equally with all conspecifics. Long-term observations of this population have shown that individuals from the same matriline almost always associate together (Bigg et al. 1990; Ford et al. 2000) and individuals from different matrilines associate less often but at varying degrees of frequency (Bigg et al. 1990; Ford and Ellis 2002). However, even association patterns between members of the same matriline are not uniform: while young offspring are mostly in very close proximity, of a few metres, of their mother (and consequently of their siblings as well), adult sons and adult daughters (plus the daughters’ offspring) can often be further away. Furthermore, proximity of matriline members varies with behavioural states such as travelling, feeding or socializing (Bigg et al. 1990).

We examined whether the three association thresholds generated different patterns of social variability at the population level as well as within matrilines. To do this, we used the coefficient of variation of association values (CV) as a metric to quantify patterns of social variation in a population (Ferreira et al. 2020). While low CV values indicate equal strengths of associations among individuals in a society, high CV values indicate variability in association strengths, where both strong and weak associations are present. For each criterion, we calculated the CV of association values and CV of non-zero association values in the whole population (since large numbers of non-existent associations can inflate the CV). The probabilistic and the encounter criteria were further compared at the matriline sublevel, by calculating CV values for matrilines with more than 5 members identified in this study, to allow for a sufficient number of dyads to be input into the analysis. To test whether CV values were significantly different from random, permutation tests were performed. For this, the data tested was randomized by permuting the associations within samples (days) 1000 times with 1000 trials (inversion of part of the matrix of associations during a sampling period) per permutation (Bejder et al. 1998; Whitehead 2008a). A Mantel test was used to test the null hypothesis of similar associations strengths within and between matrilines, with associations between categories permuted 1000 times (Schnell et al. 1985).

We expected that the same-frame threshold, being a stringent criterion, would generate few associated dyads because it will only pick out the strongest associations in the population. As a result, when focussing on the non-zero associations, it would show little social variation. In contrast, the encounter threshold was expected to generate a much larger number of associations among individuals, which might drown out the finer details of associations within each encounter and in particular within matrilines. Therefore, we expected the probabilistic method to outperform the same-frame criterion at generating patterns of social variation at the population level; and to outperform the encounter criterion at identifying social variation in associations within matrilines while generating patterns of stronger associations among individuals of the same matriline than from different matrilines.

To further explore how different association criteria can impact our perception of the social structure under study, we performed several social analyses. For each association criteria, we quantified the distribution of association index values in the population. The Newman’s (2006) eigenvector-based clustering method was used to delineate community clusters by maximizing modularity (*Q*). This top-down division technique delineates community clusters by exploring whether the dataset can be usefully divided, in such a way that associations within clusters are generally high and associations among individuals in different clusters are generally low (Whitehead 2017). Social differentiation (another measure of how diverse the associations are, where values close to 0 indicate homogeneous associations and values > 1.0 indicate diverse relationships across dyads) was estimated using the likelihood method (Whitehead 2008b). Social networks were displayed in a sociogram using the package *igraph* (Csardi and Nepusz 2006) in R 3.6.1 (R Core Team 2019).

To investigate whether different types of data and data collections should be considered separately when using the probabilistic association threshold method, we tested the robustness and stability of the social structure results in different analysis setups of the northern resident killer whale dataset (see Appendix). Each setup used the exact same dataset but the types of data within were subcategorized according to data type (film or digital photography), collection process (dedicated or non-dedicated data collection) or encounter size (see Appendix Table A1). This subcategorization aimed to test the impact of data differences on the probabilistic threshold method: data type, to reveal the effect of differences inherent to the collection system, such as photographic rates; collection process, to test the performance of the data-driven approach with a systematic vs. a more random sampling protocol; and encounter size, to test the impact of the number of animals present. For each type of data considered, associations were defined using the specific probabilistic association threshold estimated for that subset of data type (e.g., one for film photography data only and another for digital photography only).

## Results

### Probabilistic association threshold

**Fig. 2:**
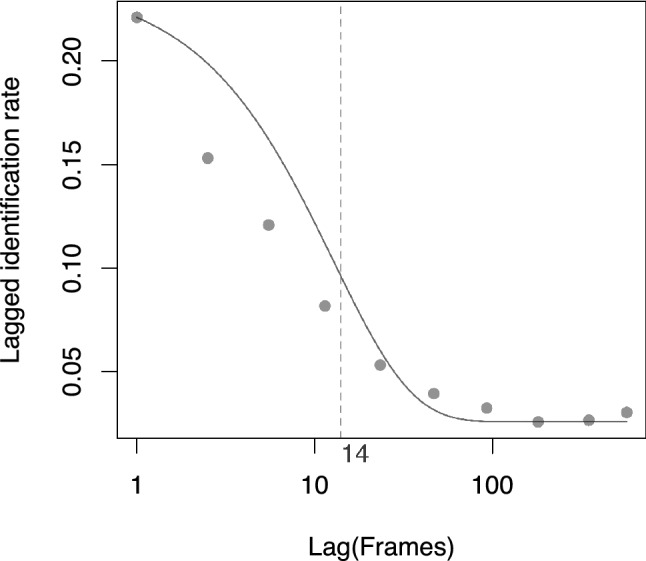
Lagged identification rate for the northern resident killer whale dataset (solid circles) against frame lag and expected lagged identification rate from a model of emigration and reimmigration (solid line). A maximum lag of 878 frames was considered. The fitted lagged identification rate estimated a mean residence time of individuals in photographable clusters of 14 frames (dashed line)

When applied to the northern resident killer whale dataset, the LIR fell quickly after individuals were photographed for the first time, with the ${\text{emigration}} + {\text{reimmigration}}$ model estimating a mean residence time in a photographable cluster of 14 frames (Fig. 2). This indicated that individuals photographed within 14 frames were likely together in the same cluster (with approximately 75% probability) and thus can be assumed to have been associating according to the probabilistic association threshold.

### Performance testing

All three criteria generated associations patterns in the population that were significantly different from random (CV permutation tests, *P* value = < 0.0001–0.001; Table 1). Furthermore, all three criteria generated dyadic association patterns that supported structuring in these associations, with stronger associations within matrilines than between matrilines (positive matrix correlation and significant Mantel tests, Table 1) but the probabilistic association showed a slightly higher matrix correlation between association strength and matriline membership. While the same-frame criterion generated a higher CV value in the population overall, the value was higher for the probabilistic association and the encounter criterion when considering the CV of non-zero associations (Table 1). As expected, when looking at the variation in association strength within matrilines, the probabilistic threshold generated association patterns that showed higher CV values than the encounter criterion (Table 2). Together, these results suggest that the social patterns produced by the probabilistic association method better captured the characteristics of the matrilineal system in the northern resident killer whale population while highlighting the expected variability in individual social preferences.

**Table 1:** Summary of social analysis results for three association criteria

	Same-frame	Probabilistic	Encounter
**Association index**			
Mean ± SD HWI	0.01 ± 0.04	0.06 ± 0.14	0.09 ± 0.17
Non-zero mean ± SD HWI	0.16 ± 0.12	0.21 ± 0.20	0.22 ± 0.21
Percentage of HWI = 0	95.3%	71.7%	57.7%
Percentage of non-zero HWI < 0.5	4.6%	24.9%	37.1%
Percentage of HWI ≥ 0.5	0.1%	3.4%	5.2%
**Coefficient of variation in associations**			
CV HWI	5.77	2.42	1.91
Permutation test, *P* value	< 0.0001	0.001	0.001
CV non-zero HWI	0.78	0.97	0.98
Permutation test, *P* value	< 0.0001	0.001	0.001
**Within vs. between matriline associations**			
Matrix correlation	0.53	0.79	0.75
Mantel test, *P* value	< 0.0001	< 0.0001	< 0.0001
**Newman’s (**2006**) clustering**			
*Q*	0.71	0.54	0.46
Clusters identified	18	9	8
Number of individuals in a cluster	1–15	4–20	7–21
**Social differentiation**			
*S*	0.64	1.11	1.09

*HWI* half-weight index of association, *SD* standard deviation, *CV* coefficient of variation, *Q* maximum modularity, *S* social differentiation

**Table 2:** Coefficients of variation in association values (CV) within the 7 matrilines included in this study with more than 5 members, for both the probabilistic association and the encounter association criteria

Matriline	Number of members	Probabilistic		Encounter	
		CV	*P* value	CV	*P* value
A10	6	0.46	0.021	0.35	1.000
C04	7	0.32	0.035	0.25	0.999
I01	6	0.27	0.060	0.09	1.000
I11	7	0.32	< 0.001	0.14	0.999
I15	9	0.27	0.024	0.19	1.000
I31	7	0.46	0.726	0.43	0.999
R05	13	0.38	0.002	0.26	0.201

For each matriline, *P* values of the CV results were generated using 1000 permutations

**Fig. 3:**
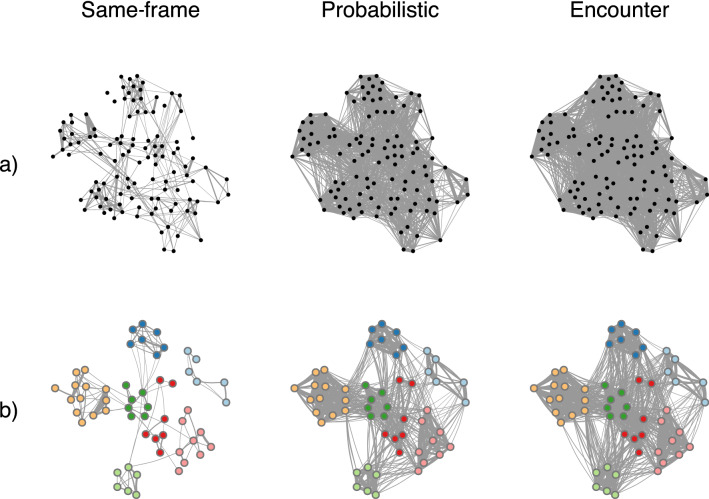
Social networks generated by the probabilistic association method and the same-frame and encounter association criteria for **a)** the 112 northern resident killer whales in the study and **b)** for the matrilines with more than 5 members. Nodes represent individuals and edges represent associations between individuals (thicker edges correspond to higher association values). For the networks of the matrilines with more than 5 members, nodes with the same colour correspond to individuals from the same matriline. Networks were plotted using Fruchterman–Reingold force-directed layout (Fruchterman and Reingold 1991) and node arrangement is kept across social networks for comparison purposes

**Fig. A1:**
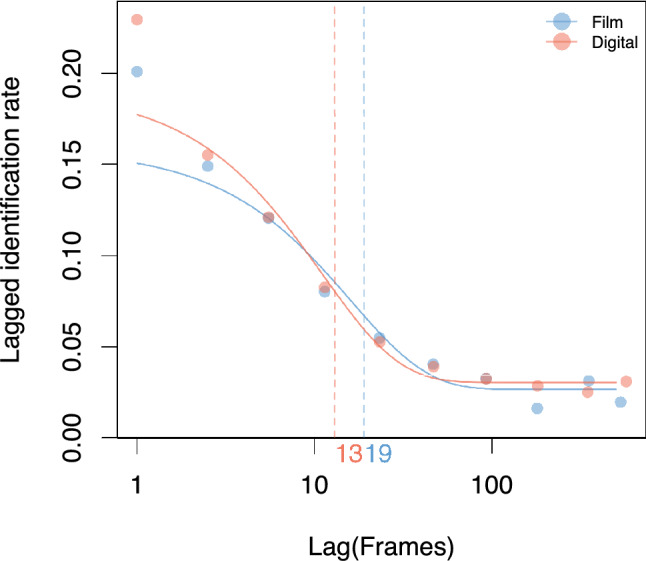
Lagged identification rates for the northern resident killer whale dataset (solid circles) against frame lag and expected lagged identification rates from a model of emigration and reimmigration (solid lines) for film (blue) and digital (coral) data separately. Maximum lags of 569 frames (film) and 878 frames (digital) were considered. The fitted lagged identification rates estimated a mean residence time of individuals in photographable clusters of 19 frames for the film data and 13 frames for the digital data (dashed lines)

**Fig. A2:**
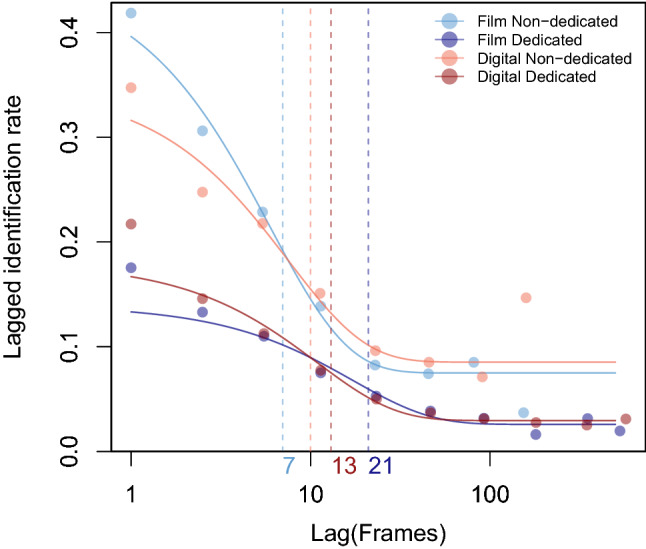
Lagged identification rates for the northern resident killer whale dataset (solid circles) against frame lag and expected lagged identification rates from a model of emigration and reimmigration (solid lines) for digital/film non-dedicated/dedicated data separately. Maximum lags of 228 frames (film non-dedicated), 569 frames (film dedicated), 255 frames (digital non-dedicated) and 878 frames (digital dedicated) were considered. The fitted lagged identification rates estimated a mean residence time of individuals in photographable clusters of 7 frames for the film non-dedicated, 21 frames for the film dedicated, 10 frames for the digital non-dedicated and 13 frames for the digital dedicated data (dashed lines)

**Fig. A3:**
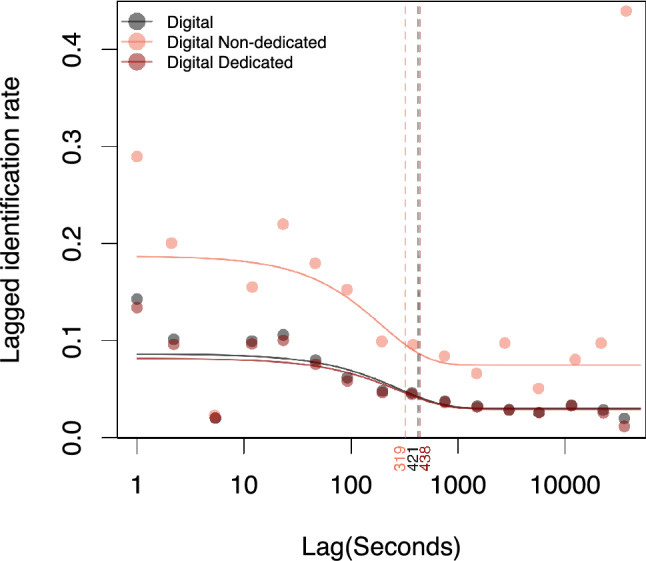
Lagged identification rates for the northern resident killer whale dataset (solid circles) against time lag in seconds and expected lagged identification rates from a model of emigration and reimmigration (solid lines) for digital, digital non-dedicated and digital dedicated data separately. Maximum lags of 41,905 s (digital), 40,327 s (digital non-dedicated) and 41,905 s (digital dedicated) were considered. The fitted lagged identification rates estimated a mean residence time of individuals in photographable clusters of 421 s for digital photography, 319 s for the digital non-dedicated and 438 s for the digital dedicated data (dashed lines). All data types present a dip in lagged identification rate data at around 5 s, possibly inherently due to the surfacing patterns of killer whales

**Fig. A4:**
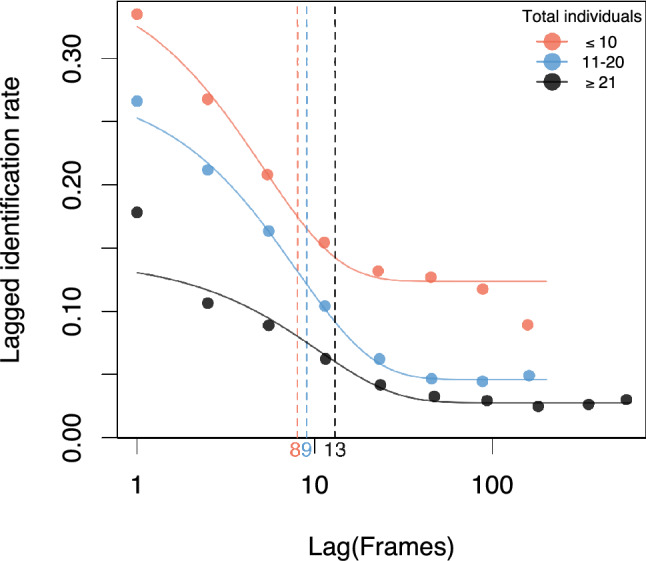
Lagged identification rates for the northern resident killer whale dataset (solid circles) against frame lag and expected lagged identification rates from a model of emigration and reimmigration (solid lines) by encounter size. Number of individuals identified in an encounter was used as a proxy for encounter size. Maximum lags of 255 frames (≤ 10 individuals), 232 frames (11–20 individuals) and 878 frames (≥ 21 individuals) were considered. The fitted lagged identification rates estimated a mean residence time of individuals in photographable clusters of 8 frames for encounters with ≤ 10 individuals identified, 9 frames for encounters with 11–20 individuals identified and 13 frames for encounters with ≥ 21 individuals identified (dashed lines)

As expected, more encompassing association criteria yielded higher numbers of associations being identified. In turn, this increased the number of existent links and resulted in stronger associations in the social networks (Fig. 3). Consequently, more encompassing association criteria resulted in an increase in association index values and a decrease in the number of clusters identified by modularity, while the social differentiation estimate generally increased (Table 1). The social network generated by the encounter criterion is somewhat cluttered with many links (i.e., associations) between individuals. In contrast, in the social network generated by the same-frame criterion, individuals are sparsely (and weakly) linked and members of the same matriline are often not connected with one another (Fig. 3). In the Appendix (Figs. A1, A2, A3, A4 and Table A2), we show that the presence of different types of data, data collection methods and number of individuals identified in an encounter produce some slight variation in association thresholds but do not alter the overall results obtained when using the probabilistic association method on this dataset.

## Discussion

We have presented a data-driven method for determining association thresholds in datasets where animals are individually identified, by estimating the probability that two individuals identified some lag value apart overlapped in close spatial proximity. We are proposing that the lag at which this probability falls to about 75% can be used as a threshold to define associations in the dataset (i.e., a high likelihood that those individuals had been in close proximity and thus associating). We described how to estimate this threshold using the lagged identification rate (LIR, Whitehead 2001) and illustrated this on a photo-identification dataset of northern resident killer whale using frame numbers as the time-dependent unit, since it was the frame-dependent information available in both the digital and the film photography data composing the dataset.

Applying the LIR approach to the test dataset showed a clear signal in the data. Lagged rates that would have remained relatively constant throughout the encounter would have yielded little information on how individuals moved in and out of the photographable cluster and associate with one another. Instead, our method estimated a probabilistic association threshold value of 14 frames. To put this into perspective, digital photographs with identified individuals that are within 14 frames of one another are on average almost 6 min apart, but with a median of 35 s (note that this estimate was only done with digital photographic data, since timestamps in seconds are not available for film photographs). Overall, this value suggests that it is quite likely (with a probability of about 75%) that individuals photographed within 14 frames of each other were together in a photographable cluster at some point during the encounter, and the likelihood of re-photographing a previously photographed killer whale substantially decreases beyond that lag. Since this data-driven method defines association based on how data are collected, this was likely either due to individuals moving out of photographic range or the photographer moving away from the grouping where the individual was present. It is important to highlight that the appropriate association threshold identified by the probabilistic technique will depend on the properties of the dataset being considered and there is no reason to assume that the specific threshold identified for the killer whale dataset tested here would be directly applicable to other datasets. Instead, we recommend that this technique be used to estimate the appropriate thresholds for each dataset.

It should be noted that the different data types in the test dataset (e.g., film vs. digital photographs, dedicated vs. non-dedicated data collections), examined in the Appendix, presented slightly different mean residence times of individuals in photographable clusters. The variation in encounter size in the film and digital photographic dataset (Figs. S2-S3 in Supplementary Material) may have caused this, since the number of individuals present impacts how the animals behave and how their behaviour is captured by the photographer. The difference in the number of photographs taken per encounter between digital and film photography does not appear to be substantial, especially for small encounters (≤ 10 individuals identified), although for larger encounters more photos seem to have been taken with digital photography and during dedicated data collection (Fig. S4 in Supplementary Material). During dedicated data collection there is a larger effort to photograph all individuals present during the observation period than when doing non-dedicated data collection, which will likely lead to more photographs being taken per encounter. This might explain the pattern, seen in both film and digital data, of a higher probabilistic association threshold (estimated using the LIR) for the dedicated data collection vs. the non-dedicated data collection (Appendix Fig. A2). This difference is more pronounced in film photography, possibly due to the limitations of the equipment and its impact on the number of times individuals are re-photographed during data collection. Photographers can assess the quality of each frame upon collection when shooting digital, but not when shooting film. Therefore, they might stay for longer with the same individuals during film photography and re-photograph them more than they would shooting digital, to ensure full coverage of the grouping. This could explain the slightly higher estimated probabilistic association threshold for film photography vs. digital photography (Appendix Fig. A1). However, overall social structure results were very similar across the data setups tested, and data subdivision by type of data did not improve the performance of the probabilistic association method when investigating the social structure of this population.

We then evaluated the performance of the probabilistic association in generating expected social patterns of the northern resident killer whales, in comparison with two arbitrary association criteria: i.e., associated if present in the same frame and associated if present in the same encounter. The probabilistic association performed better at finding the high and non-random social diversity expected at the population level (higher CV of non-zero HWI in the population and significant CV permutation test; Table 1), as well as the finer social diversity within matrilines (higher CV of HWI within matrilines; Table 2), while identifying the pattern of stronger associations among individuals within the same matriline than between individuals from different matrilines (positive matrix correlation between association strength and matriline membership, and significant Mantel test; Table 1). Social structure results and network visualisation suggest that the probabilistic method includes associations occurring in close spatial proximity, e.g., visual contact or touching (as the same-frame criterion), while limiting most distant associations (those that would have been included in the more encompassing encounter criterion). In practice, these associations happen at spatial distances of individuals forming clusters in the field (i.e., clustering individuals are in closer proximity to each other than with members of other clusters around them).

The number of community clusters delineated by the Newman’s (2006) eigenvector-based clustering method was lower than the number of different matrilines in the dataset (28 matrilines) for all three criteria, with many matrilines being clustered together. This is not surprising since, although the overall multilevel society of the northern resident killer whales remains stable, there are annual fluctuations of association patterns between and within matrilines (Ford and Ellis 2002; Stredulinsky et al. 2021). Therefore, our test dataset, comprising of four different years of data, will identify the overall stable social structure but may not be sufficient to distinguish subtle social divisions in the population.

The variation in social structure results, generated by the different association criteria, highlights how the choice of association thresholds affects our perception of social patterns and why this decision should not be taken lightly in any social study. However, this is not to say that arbitrary association thresholds are inherently flawed and should never be used. Decisions on association criteria can be made using a priori (biologically meaningful) knowledge about a social system, if these are independent from the hypothesis later tested with the created social network (Ferreira et al. 2020). For example, in the northern resident killer whale population, encounter criterion could be used to investigate matriline level dynamics and temporal patterns, because matrilines in this population are stable over long periods of time. However, this requires an underlying understanding of the social structure of a population being studied, which is missing for many social systems. Presently, the impact of using arbitrary association thresholds on our perception of social structure is mostly unknown. Using more than one definition of association when collecting data and/or running analyses, can be a way to expand our understanding of social dynamics, and examine the effects of different association definitions (e.g., Gero et al. 2015).

### Considerations and recommendations on using the probabilistic association technique

The association technique described here addresses a crucial decision-making step in any social structure study: how do we define associations among individuals to best represent true instances of interactions and to most accurately model the true social structure of the population? This technique uses the presence/absence of the individuals recorded in the data to define a threshold for the duration within which individuals are likely “together” (and in close proximity) in a grouping, thus likely associating. The main assumption is that proximity is related to social associations between individuals. The method was illustrated here on a cetacean photo-identification dataset, with cetaceans being prime candidates for this kind of methodological approach due to the repetitive nature of photographs of individuals surfacing together. Nonetheless, this technique could be applied to a wide range of datasets where conspecifics are individually identified in a sequential order that reflects spatial and temporal proximity. These include PIT-tag identifications using static readers (e.g., from tagged Gould’s wattled bats, *Chalinolobus gouldii*, Godinho et al. 2015) and acoustic individual identifications using fixed or mobile microphones or hydrophones (e.g., from grey reef sharks, *Carcharhinus amblyrhynchos*, tagged with acoustic transmitters, Jacoby et al. 2016).

The probabilistic association is a dyadic association method, since associations in a sampling period are defined using the “likely associated” threshold, dyad by dyad. In certain populations, dyadic association methods can detail associations over shorter periods of time and spatial scales than the ones where groups are enumerated. While associations by group membership (i.e., all individuals in the same group, defined in some spatiotemporal manner, are associated) would consider all individuals seen in the same group equally associated, dyadic association methods are able to distinguish unequal associations among individuals that could be present in that same group (Johnston et al. 2017). This advantage can be particularly important for populations where groups can be large, poorly delineated, long-lasting, or spread over larger areas (e.g., short-finned pilot whales, *Globicephala macrorhynchus*, off Hawai‘i Island, Mahaffy et al. 2015), in which individuals at one end of the group might not necessarily be interacting with individuals at the other end.

Some other advantages of this data-driven method are that: (1) it simplifies data collection in the field, since it does not require concurrent collection of behavioural or group information alongside identifications; (2) it is more objective, since it does not involve the in-situ identification of spatiotemporal groups, which can be especially problematic when groups are so subtle, dynamic or fluid that they cannot be rigorously distinguished by observers (e.g., Icelandic killer whales, Tavares et al. 2017). This not only removes observer bias when groups are apparently discernable, but also makes this method widely applicable to populations/species that present high social dynamics, and across different behavioural states (which can change how groupings are perceived by observers); (3) to some degree, it allows for association analysis from any identification dataset, even if it was not collected for the purposes of studying social structure (e.g., without concurrent group membership information); and (4) it allows for different types of identification data (such as the ones obtained using film or digital photography, or PIT-tag readers with different properties) to be used jointly in the analysis. Indeed, the fact that not all data need be collected in dedicated studies with the same protocol and equipment, and detailed recording of timestamps of identifications, is the major advantage of this method over other data-driven techniques (e.g., Gaussian mixture model time windows of tagged individuals, Psorakis et al. 2015; maximum-likelihood estimation of photographic bouts, Tavares et al. 2017).

As a data-driven method, the limitations of the methodology are inherently connected to the quality of the data collection. The most common bias in a dataset occurs when not all associates of an individual are identified, which can happen when associates are not seen by the observer or are not identified while associating with that particular individual (and this physical proximity is not recorded). This is more prominent in populations where some individuals are more easily recognizable than others, due to differences in individuals’ distinctive characteristics (Whitehead 2008a). Since this bias can affect how individuals are represented in the dataset, the best way to account for it when doing social structure analyses is to use an association index that can correct for some of the bias (Whitehead 2008a; Whitehead and James 2015) or to use permutations to build null models that account for these kinds of sampling differences (Farine and Whitehead 2015). Additionally, data collection in situations where multiple groupings of animals are seen in a relatively small area, or populations/species exhibit high social dynamics, can be more challenging. Even in situations like these, it is reasonable to assume that the majority of identifications collected in close sequence are of individuals present in the same general area (i.e., photographable cluster), since the sampling range will be restricted by the researcher’s spatial positioning and equipment used. However, as for any other association threshold criteria, there is always the possibility that some of the individuals considered associated were not truly associating. With substantial datasets, this error should be diluted and should not be an issue, unless inferences are made at the dyadic level based upon limited data. Finally, inconsistencies in sampling effort and performance (e.g., photographic collection rate) across data collection events could introduce noise to the dataset. Potential limitations should be recognised and considered individually for each study, but with sufficient sampling effort and large datasets the effect is likely to be negligible.

Based on our tests, we can tentatively make several points of advice for the use of the methodology described in this study. When sampling, we recommend that the protocol be in line, as much as possible, with the assumption that individuals photographed chronologically closer were also closer in space (Johnston et al. 2017), since an ordinal unit of time (direct, like real time in seconds, or indirect, like sequential frame numbers) associated with the identifications, is used as a proxy for spatial distance. From the analysis performed on the test dataset, we suggest that the best approach is to calculate lags between identifications using an ordinal parameter that is chronological and common to all data in the dataset (such as frame numbers, when not all photographs have associated timestamps). It is appropriate to consider whether the data should be divided into types, particularly the ones resulting from sampling discrepancies across data collection events (e.g., due to the use of different sampling equipment). The level of variation in mean residence times in a photographable cluster (i.e., probabilistic association threshold) across the different data types should be tested, and how this variation might affect subsequent results should be assessed. In the subsequent social analysis, the full dataset should be analysed without it being subdivided into types of data, unless substantial differences in mean residence times are found in one or more data types. In this case, subdividing the data and using subset-specific mean residence times is likely best practice, since it could noticeably affect final results. For datasets where real time of sampling was registered for all identifications, using real time as the ordinal parameter for lag calculation could theoretically give a finer resolution of associations. This was not observed in our test dataset, where no considerable differences were seen in results when using real time rather than frame number for the digital data (see Appendix Table A2). However, this could be a result of the specific social structure of this population and cannot be generalised to other populations/species with different social dynamics.

## Conclusion

We have shown that the choice of association threshold can affect the estimated social patterns. Therefore, the way we define associations for the analysis of a population’s social structure is an important step that requires careful consideration. To address this, we presented a widely applicable, data-driven approach for finding association thresholds using sequential identifications of distinctive individuals as a proxy for distance among them. Using data-driven approaches to define associations should minimize the risk of skewing estimated social structures towards specific preconceived social patterns, while potentially overlooking other patterns that are not obvious during data collection.

### Electronic supplementary material

Below is the link to the electronic supplementary material.

Supplementary file1 (PDF 168 KB)

Supplementary file2 (PDF 171 KB)

## Data Availability

The datasets analysed in the current study are available from Thomas Doniol-Valcroze (Thomas.Doniol-Valcroze@dfo-mpo.gc.ca) on reasonable request.

## Appendix

In this Appendix, we test the applicability of the probabilistic association threshold methodology to a dataset with different types of data (and data collections) by investigating whether those should be considered separately when using this method. First, we estimated probabilistic association thresholds separately for the following subsets of the northern resident killer whale dataset, using photographic frame numbers as the ordinal parameter: (1) film photography; (2) digital photography; (3) non-dedicated film photography; (4) dedicated film photography; (5) non-dedicated digital photography; (6) dedicated digital photography; (7) encounters with ≤ 10 individuals identified; (8) encounters with 11–20 individuals identified; and (9) encounters with ≥ 21 individuals identified. For the digital photography, the non-dedicated digital photography and the dedicated digital photography subsets of the data, we also calculated probabilistic association thresholds using the photographs’ timestamps (to the second) as the ordinal parameter.

The values estimated from the LIR model fitting indicated decreasing probability at slightly higher frame numbers for film than for digital (film versus [vs.] digital: 19 vs. 13 frames, Fig. A1). Frame association thresholds were slightly lower for non-dedicated data collections than for dedicated ones (digital photography, non-dedicated vs. dedicated: 10 vs. 13 frames; film photography, non-dedicated vs. dedicated: 7 vs. 21 frames, Fig. A2). The values of time in seconds across digital photography types were fairly high (319–438 s, Fig. A3), which could have resulted from the longer data tail of these data (in comparison with the frame number ones) driving the results slightly up. The results of the model fitting when considering encounter sizes were very similar across size ranges (8–13 frames, Fig. A4).

We then tested the stability of the social structure results by measuring associations in different setups of the northern resident killer whale dataset: individuals were considered associated for the day (sampling period) if photographed within the value identified by the LIR analyses for that specific data type being considered in the setup (Table A1). For example, in the film/digital setup we identified the data as either from film photography or from digital photography. For each setup, association patterns were tested as described in the Methods section. Results of the social structure analyses were overall similar between the ones obtained without data distinctions (probabilistic association presented in the Results section) and the different setups tested (Table A2). Associations were different from random for all setups (CV permutation tests, *P* value = < 0.001–0.001) and CV values, the distribution of association indices, patterns of dyadic association within vs. between matrilines and social differentiation were overall similar across setups. The number of clusters delineated by maximizing modularity were identical across most setups (Table A2) but the setups grouped some of the matrilines in different clusters. The encounter sizes setup found more clusters in the dataset (Table A2), only due to variation in how many matrilines were separated from others in a single cluster (while some clusters were larger and grouped more matrilines together). Overall, the presence of different types of data, data collection and sizes of encounters (i.e., varying number of individuals that might be present in an encounter) did not seem to hinder the use of this methodology.

**Table A1:** Analysis setups tested and correspondent probabilistic association criteria (see Figs. A1, A2, A3, A4 for lagged identification rate curves of the different types of data considered)

Analysis setup	Probabilistic association threshold used
Film/digital	Individuals associated if seen within 19 frames of each other in film photography and within 13 frames in digital photography
Film/digital non-ded./ded	Individuals associated if seen within 7 frames of each other in non-dedicated film photography, 21 frames in dedicated film photography, 10 frames in non-dedicated digital photography and within 13 frames in dedicated digital photography
Film/digital seconds	Individuals associated if seen within 19 frames of each other in film photography and within 421 s in digital photography
Film/digital non-ded./ded. seconds	Individuals associated if seen within 7 frames of each other in non-dedicated film photography, 21 frames in dedicated film photography, 319 s in non-dedicated digital photography and within 438 s in dedicated digital photography
Encounter sizes	Individuals associated if seen within 8 frames of each other in encounters with ≤ 10 individuals identified, 9 frames in encounters with 11–20 individuals identified, and within 13 frames of each other in encounters with ≥ 21 individuals identified

*Non-ded.* non-dedicated, *Ded.* dedicated

**Table A2:** Summary of results calculated using probabilistic association thresholds on the five different data setups tested (see Table A1)

	Film/digital	Film/digital non-ded./ded	Film/digital seconds	Film/digital non-ded./ded. seconds	Encounter sizes
**Association index**					
Mean ± SD HWI	0.06 ± 0.14	0.06 ± 0.14	0.05 ± 0.13	0.05 ± 0.13	0.05 ± 0.13
Non-zero mean ± SD HWI	0.20 ± 0.20	0.20 ± 0.20	0.20 ± 0.18	0.20 ± 0.18	0.20 ± 0.19
Percentage of HWI = 0	71.0%	71.2%	73.5%	73.3%	73.3%
Percentage of non-zero HWI < 0.5	25.6%	25.4%	23.8%	24.0%	23.6%
Percentage of HWI ≥ 0.5	3.4%	3.4%	2.7%	2.7%	3.1%
**Coefficient of variation in associations**					
CV HWI	2.38	2.38	2.47	2.46	2.49
* P* value	0.001	0.001	< 0.0001	0.001	< 0.0001
CV non-zero HWI	0.97	0.96	0.94	0.94	0.95
* P* value	0.001	0.001	< 0.0001	0.001	0.001
**Within vs. between matriline associations**					
Matrix correlation	0.79	0.78	0.77	0.77	0.77
Mantel test, *P* value	< 0.0001	< 0.0001	< 0.0001	< 0.0001	< 0.0001
**Newman’s (**2006**) clustering**					
* Q*	0.54	0.54	0.53	0.53	0.54
Clusters identified	9	9	9	9	11
Number of individuals in a cluster	4–19	4–20	6–19	6–19	3–19
**Social differentiation**					
* S*	1.10	1.10	1.08	1.07	1.09

*HWI* half-weight index of association, *SD* standard deviation, *CV* coefficient of variation, *Q* maximum modularity, *S* social differentiation
