# Supplementary material for: Assessing social structure: a data-driven approach to define associations between individuals
Source: Mamm Biol. 2022 Mar 25;102(3):551–66. doi: 10.1007/s42991-022-00231-9 (PMC9883313; doi:10.1007/s42991-022-00231-9)
Supplement: Supplementary file 1 — Supplementary file1 (PDF 168 KB) [file 42991_2022_231_MOESM1_ESM.pdf]

Supplementary Material

Assessing social structure: a data-driven approach to define associations between individuals

Sara B. Tavares[1,*], Hal Whitehead[2], Thomas Doniol-Valcroze[1]

[1] Cetacean Research Program, Pacific Biological Station, Fisheries and Oceans Canada, Nanaimo, Canada
[2] Department of Biology, Dalhousie University, Halifax, Canada
[*] Corresponding author's e-mail address: sarabrito.tavares@gmail.com. Corresponding author's ORCID: 0000-0001-7216-6913

Figure	Page number in Supplementary Material
**S1**	2
**S2**	3
**S3**	4
**S4**	5

Mammalian Biology **(Special Issue) 102 (3)**
Individual Identification and Photographic Techniques in Mammalian Ecological and Behavioural Research – Part 1: Methods and Concepts

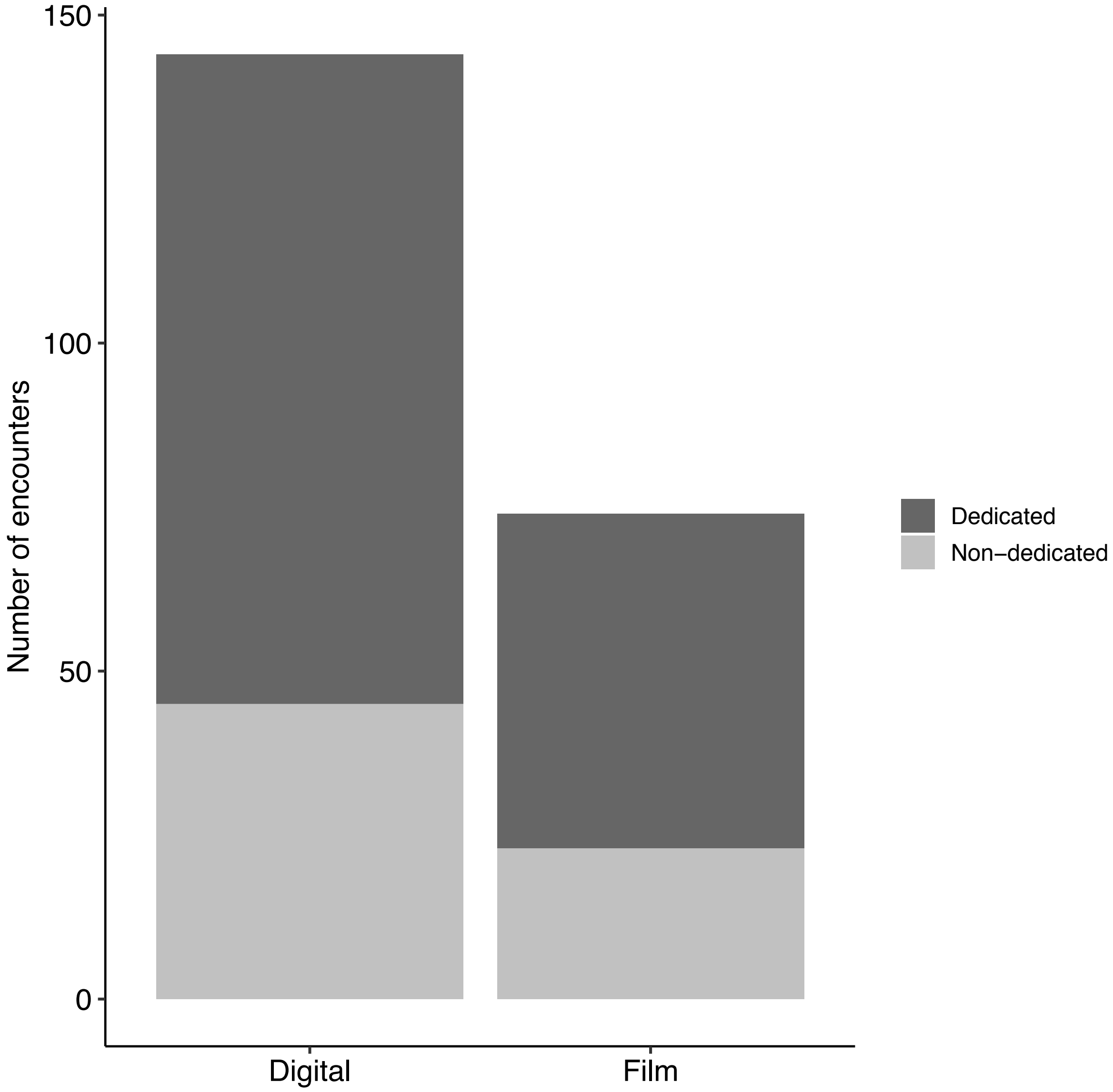

Fig. S1 Number of encounters in digital and film photography data by type of data collection (dedicated or non-dedicated)

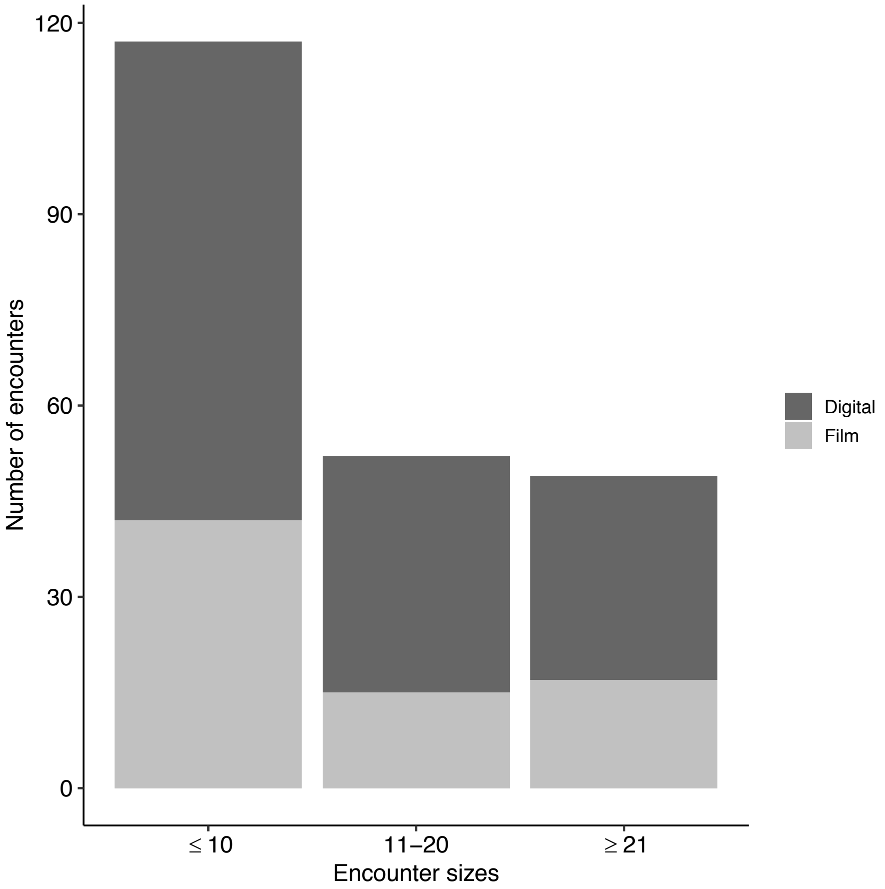

Fig. S2 Number of encounters per encounter size when using either digital or film photography. Number of individuals identified in an encounter was used as a proxy for encounter size

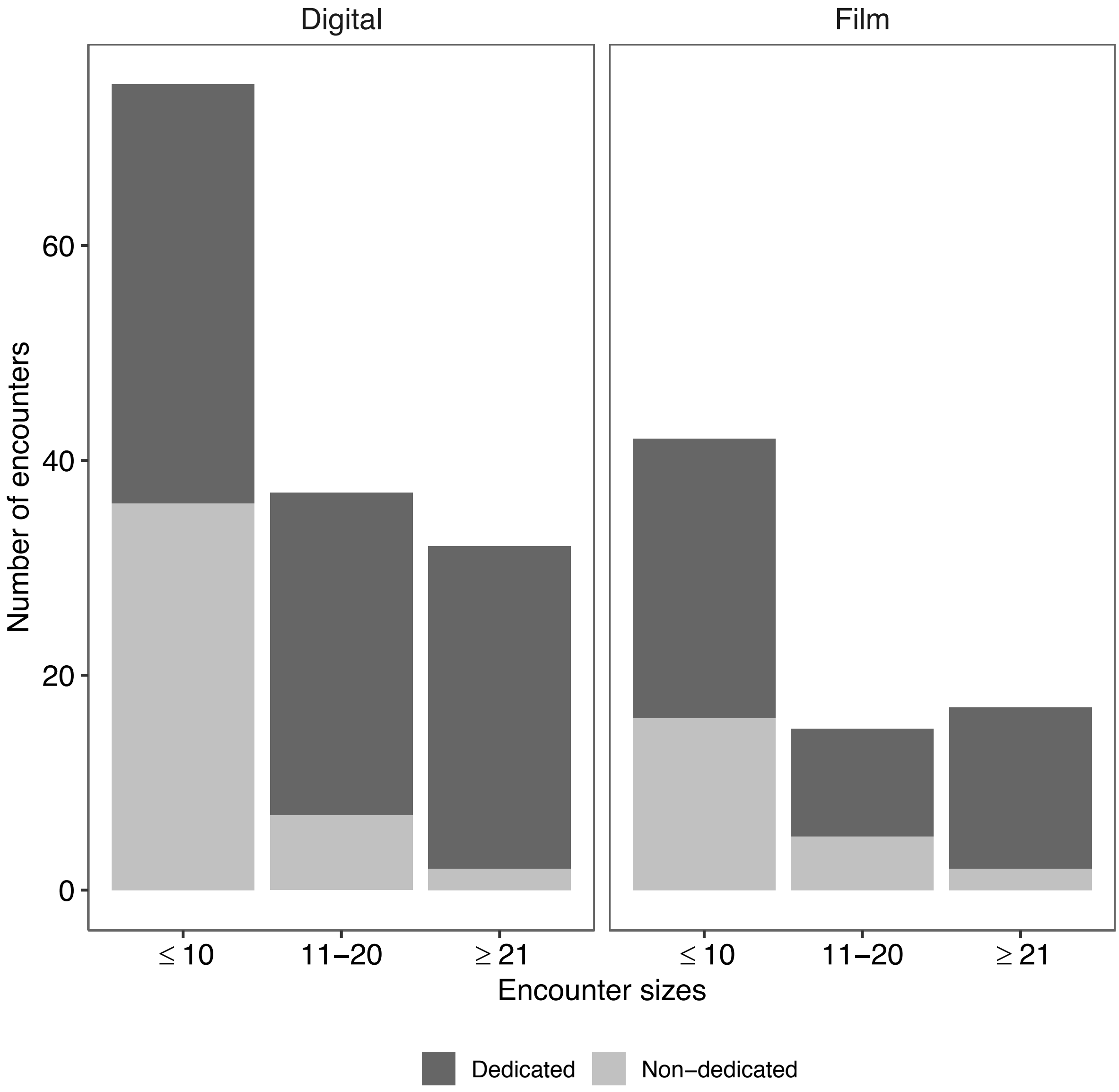

Fig. S3 Number of encounters per encounter size in digital and film photography data by type of data collection (dedicated or non-dedicated). Number of individuals identified in an encounter was used as a proxy for encounter size

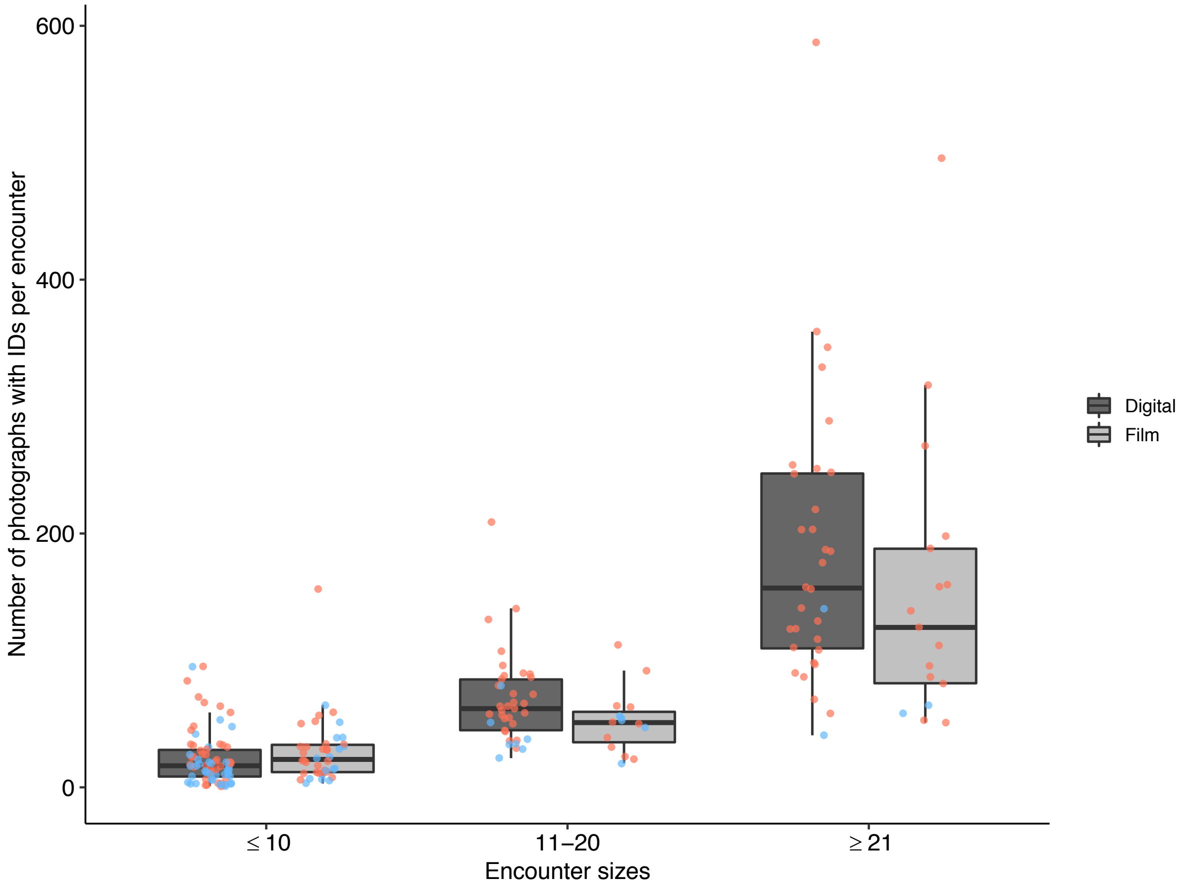

Fig. S4 Number of photographs taken in an encounter, with at least one identified individual, per encounter size, in digital and film photography data. Boxplots show individual observations (coral dots for dedicated data collections and blue dots for non-dedicated data collections), median (horizontal bar), 1st and 3rd quartiles (lower and upper hinges, respectively), lower and largest values within 1.5 times the distance between the 1st and 3rd quartiles (lower and upper whiskers, respectively). Number of individuals identified in an encounter was used as a proxy for encounter size
