# Supplementary material for: Assessing social structure: a data-driven approach to define associations between individuals
Source: Mamm Biol. 2022 Mar 25;102(3):551–66. doi: 10.1007/s42991-022-00231-9 (PMC9883313; doi:10.1007/s42991-022-00231-9)
Supplement: Supplementary file 2 — Supplementary file2 (PDF 171 KB) [file 42991_2022_231_MOESM2_ESM.pdf]

Supplementary Material no. 2

Assessing social structure: a data-driven approach to define associations between individuals

Sara B. Tavares*, Hal Whitehead, Thomas Doniol-Valcroze

* sarabrito.tavares@gmail.com

Highlights:

- An approach is proposed for defining association thresholds on photo-identification datasets
- The approach uses lagged identification rates and a simple model of emigration/immigration from photographable clusters to identify a probabilistic association threshold
- The association threshold is a time-ordered lag value between identifications of two individuals that corresponds to a ~75% probability they were in close spatial proximity and likely associating
- The approach was tested on a photographic dataset of northern resident killer whales (*Orcinus orca*) in the Northeast Pacific
- The proposed approach captured social patterns at different structural levels better than two other arbitrary thresholds

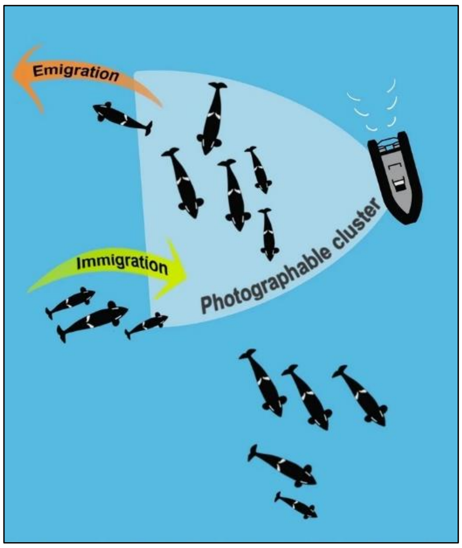

This article is part of a thematic collection of articles (Special Issue) of *Mammalian Biology* and covers the following topics and taxa (marked with ☑) addressed in the Special Issue:

Article Type

☐ Original Research	☑ Techniques	☐ Review	☐ Short Communication	☐ Concept Note

Taxon

Terrestrial

☐ Bats *(Order Chiroptera)*	☐ Primates : Great Apes *(Family Hominidae)*
☐ Carnivores : Bears *(Family Ursidae)*	☐ Primates : Old World monkeys *(Family Cercopithecidae)*
☐ Carnivores : Canids *(Family Canidae)*	☐ Ungulates : Bovids *(Family Bovidae)*
☐ Carnivores : Felids *(Family Felidae)*	☐ Ungulates : Deers *(Family Cervidae)*
☐ Carnivores : Hyenas *(Family Hyaenidae)*	☐ Ungulates : Giraffes *(Family Giraffidae)*
☐ Carnivores : Mustelids *(Family Mustelidae)*	☐ Ungulates : Horses *(Family Equidae)*
☐ Elephants *(Family Elephantidae)*	☐ Multiple taxa *(3 or more Families/Orders)*

Marine

☐ Baleen whales : Right whales *(Family Balaenidae)*	☑ Large toothed whales *(Families Delphinidae & Hyperoodontidae)*
☐ Baleen whales : Rorquals *(Family Balaenopteridae)*	☐ Pinnipeds : True seals *(Family Phocidae)*
☐ Carnivores : Bears *(Family Ursidae)*	☐ Porpoises *(Family Phocoenidae)*
☐ Carnivores : Mustelids *(Family Mustelidae)*	☐ Sirenians : Manatees *(Family Trichechidae)*
☑ Dolphins *(Family Delphinidae)*	☐ Multiple taxa *(3 or more Families/Orders)*

Topic

☐ Acoustic ID	☐ Identification techniques
☐ Aerial surveys	☐ Life-history
☑ Analytical innovations	☐ Machine learning
☐ Automated pattern recognition	☐ Mark-recapture analysis
☐ Behavioural ecology	☐ Morphometrics
☐ Camera-trapping	☑ Network analysis
☐ Conservation management	☐ Photogrammetry
☐ Data management	☐ Population ecology
☐ Demographic parameters	☐ Site fidelity & Movement
☐ Field methodology	☑ Social ecology
☐ Genetic ID	☐ Software/Package development
☐ Health conditions	☐ Thermal imagery
☐ Other: *(please specify)*	

References

Karczmarski L, Chan SCY, Rubenstein DI, Chui SYS, Cameron EZ (2022a). Individual identification and photographic techniques in mammalian ecological and behavioural research – Part 1: Methods and concepts. *Mammalian Biology* (Special Issue), 102 (3) https://link.springer.com/journal/42991/volumes-and-issues/102-3

Karczmarski L, Chan SCY, Chui SYS, Cameron EZ (2022b). Individual identification and photographic techniques in mammalian ecological and behavioural research – Part 2: Field studies and applications. *Mammalian Biology* (Special Issue), 102 (4) https://link.springer.com/journal/42991/volumes-and-issues/102-4
